# The Role of mRNA Modifications in Bone Diseases

**DOI:** 10.7150/ijbs.104460

**Published:** 2025-01-13

**Authors:** Zehui Li, Keyu Meng, Shanwei Lan, Zhengda Ren, Zhongming Lai, Xiang Ao, Zhongyuan Liu, Jiajia Xu, Xiaoyi Mo, Zhongmin Zhang

**Affiliations:** Division of Spine Surgery, Department of Orthopaedics, Nanfang Hospital, Southern Medical University, Guangzhou, Guangdong 510515, China; The First School of Clinical Medicine, Southern Medical University, Guangzhou, Guangdong 510515, China.

**Keywords:** messenger RNA, RNA modifications, bone diseases

## Abstract

As a type of epigenetic modifications, mRNA modifications regulate the metabolism of mRNAs, thereby influencing gene expression. Previous studies have indicated that dysregulation of mRNA modifications is closely associated with the occurrence and progression of bone diseases (BDs). In this study, we first introduced the dynamic regulatory processes of five major mRNA modifications and their effects on the nucleus export, stability, and translation of mRNAs. We then summarized the mechanisms of mRNA modifications involved in the development of osteoporosis, osteoarthritis, rheumatoid arthritis, ankylosing spondylitis, fractures, osteomyelitis, and osteosarcoma. Finally, we reviewed therapeutic strategies for BDs based on the above mechanisms, focusing on regulating osteoblast and osteoclast differentiation, inhibiting cellular senescence and injury, and alleviating inflammation. This review identified mRNA modifications as potential targets for treating BDs and proposes perspectives on the diversity, targetability, and safety of mRNA-modifying therapies.

## 1. Introduction

Gene expression is influenced by epigenetic modifications without altering nucleotide sequences 1. With the rapid advancement of epigenetics, research has expanded beyond traditional DNA and histone modifications to encompass RNA modifications—a diverse and complex class of epigenetic modifications with over 170 types identified to date 2. Among all RNAs, mRNA carries genetic information from DNA and serves as a template for protein synthesis. mRNA modifications regulate mRNA metabolism and affect gene expression. Recently, mRNA modifications have become a research hotspot for a wide range of diseases, especially cancer, neurodegenerative diseases, and autoimmune diseases 3-5.

An increasing number of studies have focused on mRNA modifications that regulate bone diseases (BDs). m6A, ac4C, and m5C modifications are shown to regulate the metabolism of mRNAs related to osteoclastic and osteogenic differentiation, and they then participate in the occurrence and development of osteoporosis (OP) 6-8. In rheumatoid arthritis (RA), abnormalities in m5C and m6A modifications of related mRNAs have been reported to exacerbate joint inflammation and the proliferation of fibroblast-like synoviocytes (FLSs) 9, 10. The proliferation and metabolic abnormalities observed in osteosarcoma (OS) cells can be linked to dysregulated mRNA modifications, specifically in m6A, m5C, and ac4C 11-13. In addition, numerous studies have shown that mRNA modifications are also involved in the development of fractures, osteoarthritis (OA), osteomyelitis (OM), and ankylosing spondylitis (AS). Thus, abnormal mRNA modifications disrupt normal cellular functions, contributing to the development and progression of BDs.

Based on the above mechanisms of mRNA modifications, researchers have proposed corresponding therapeutic strategies for BDs. Viruses and certain drugs have been used to regulate mRNA modification regulators 6, 8, 14. Specifically, adenovirus, lentivirus, and adeno-associated virus are used as vectors to elevate the levels of METTL14 (an m6A writer) and YTHDF1 (an m6A reader) in bone marrow mesenchymal stem cells (BMSCs), which contribute to increased osteoblast differentiation by enhancing the stability and translation of autophagy genes such as *Beclin-1*, ultimately alleviating bone loss in OP and accelerating bone healing in fractures 6, 14. The m5C reader Y-box binding protein 1 (YBX1) is activated by sciadopitysin, resulting in increased bone mass in ovariectomized (OVX) mice by bolstering the stability of bone morphogenetic protein 4 (*BMP4*) 8. These studies suggest that therapies targeting mRNA modifications could correct abnormal cellular functions and prevent the progression of BDs.

In this review, we introduced the dynamic regulatory mechanisms of five major mRNA modifications and their effects on mRNA metabolism (nucleus export, stability, translation, etc.). We then summarized the pathogenesis of the mRNA modifications involved in BDs, including fracture, OP, OA, RA, AS, OM, and OS. Finally, we concluded the strategies of mRNA-modifying therapies for BDs, mainly including the regulation of osteoblast and osteoclast differentiation, as well as the inhibition of cell senescence, injury, and inflammation. This review describes the cross-talk between mRNA modifications and BDs, which may uncover novel insights into BD mechanisms and pave the way for the development of epigenetic-based therapeutic approaches for BDs.

## 2. Overview of mRNA modifications (Fig. 1)

The understanding of mRNA modifications is gradually deepening with advanced detection technologies. Currently, over 170 types of RNA modifications have been discovered across various RNA species. Chemical modifications play important roles in the metabolism and function of mRNA, thus widely regulating human diseases. The regulators of mRNA modifications primarily consist of writers, erasers, and readers. Writers install chemical modifications on RNAs, while erasers remove these modifications to ensure the dynamic reversibility of modifications. Readers, on the other hand, recognize modification sites, bind to the modified RNAs, and regulate their metabolism and function. In this review, we focused on five major mRNA modifications closely related to BDs and summarized how these modifications regulated the metabolism of mRNAs through regulators.

### 2.1 N6-methyladenosine (m6A) (Fig. 1a)

m6A, a chemical modification resulting from the addition of a methyl group to the sixth nitrogen atom of adenosine, was initially discovered by Saneyoshi *et al.* in the valine tRNAs of *Escherichia coli* in 1969 15. With the emergence of technologies such as MeRIP-seq 16, m6A modifications have been discovered to be prevalent in mammalian mRNAs, and their abundance undergoes dynamic changes during mammalian development 17. The m6A modifications of mRNA are primarily catalyzed by the methyltransferase complex (MTC), with METTL3, METTL14, and WTAP serving as core subunits 18. METTL3 exhibits methyltransferase activity, whereas METTL14 and WTAP exert pivotal influences on m6A levels through their supporting functions 19. METTL14 promotes the allosteric activation of METTL3, facilitating the recognition of RNA substrates 20. WTAP mediates the localization of the METTL3-METTL14 complex to nuclear speckles and significantly influences their accumulation within these nuclear speckles 21. In mammalian mRNAs, m6A modifications cluster near the stop codon and 3' untranslated region (3' UTR), often within the GGACU sequence 22. This localization is likely related to the recruitment of the MTC to specific mRNA regions by VIRMA, RBM15, and RBM15B 23. Moreover, the occurrence of m6A modifications in different regions of mRNA may influence mRNA metabolism in varying ways, contributing to diverse effects.

Currently, the identified m6A erasers primarily consist of fat mass and obesity-associated protein (FTO) and ALKBH5, which achieve the reversibility of m6A modifications 24. FTO has been shown to remove m6A modifications from nuclear mRNAs by catalyzing their demethylation using α-ketoglutarate 25. ALKBH5, on the other hand, catalyzes the demethylation of m6A through a Fe^2+^-dependent mechanism 26.

Typical m6A readers are members of the YTH domain-containing protein family, which directly recognizes m6A sites on mRNAs through their YTH domains, thus regulating mRNA metabolism 27. YTHDC1 resides in the nucleus, regulating mRNA splicing and promoting nuclear export 28. The YTHDC2 protein is localized in both the nucleus and cytoplasm, where it plays a role in mRNA degradation by recruiting the 5'→3' exoribonuclease XRN1 29. However, another study demonstrated that YTHDC2 has a relatively modest effect on mRNA stability and, more notably, can enhance translation efficiency 30. The mechanism of YTHDC2 in regulating mRNA translation and stability needs to be further explored. It has been reported that YTHDF2 removes mRNAs from the translatable pool, thereby promoting their degradation 31, while YTHDF1 promotes translation by recruiting translation initiation factors and facilitating ribosome loading onto mRNA 32. In addition, YTHDF3 promotes protein synthesis in synergy with YTHDF1 and affects the decay of methylated mRNAs mediated by YTHDF2 33. Insulin-like growth factor 2 mRNA-binding proteins (IGF2BPs) and heterogeneous nuclear ribonucleoproteins (hnRNPs) also serve as m6A readers 34, 35. hnRNPs mainly regulate the processing of pri-miRNAs and pre-mRNAs 36, 37, while IGF2BPs enhance mRNA stability and translation by recruiting RNA stability factors and eukaryotic translation initiation factor (eIF) proteins 34, 38. In summary, the differential expression of m6A readers in various biological contexts may lead to distinct outcomes of mRNA modifications, thereby playing opposing roles in the onset and progression of diseases.

### 2.2 N1-methyladenosine (m1A) (Fig. 1b)

m1A is a methylation modification that occurs on the first nitrogen atom of RNA adenine. Since its initial discovery in 1961, m1A modifications have primarily been identified in the tRNAs and rRNAs of various fungi and bacteria 39, 40. In 2016, Dominissini *et al.* demonstrated the existence of m1A modifications in eukaryotic mRNAs using MeRIP-seq technology 41. In eukaryotic mRNAs, the ratio of m1A/A is shown to be lower compared to m6A/A and Ψ/U in the same sample. m1A is enriched near the start codon within the 5' untranslated region (5' UTR) and within the coding sequence (CDS) 42. As m1A writers, RNA methyltransferase 61A (TRMT61A) exhibits enzymatic activity, while tRNA methyltransferase 6 (TRMT6) is responsible for substrate selection 43. They form an α2β2 heterotetramer complex, TRMT6/61A, which catalyzes the m1A modifications in mRNAs 44. Additionally, TRMT61B and TRMT10C have also been reported to be involved in the m1A modifications of mRNAs 45, 46. The erasers of m1A include the AlkB family, namely ALKBH1, ALKBH3, and FTO 46. It has been reported that m6A readers (YTHDF proteins) can also bind to m1A modifications and function as m1A readers 47. The effects of m1A modifications on mRNA metabolism are position dependent. Specifically, m1A located at the 5' UTR, with its positive charge, influences the secondary/tertiary structure of mRNA around the translation initiation site and disrupts the formation of hydrogen bonds, thus interfering with Watson-Crick base pairing and promoting the translation initiation and the recruitment of elongation factors 41, 42. Moreover, m1A modifications within CDS have been reported to affect translation elongation and potentially reduce translation efficiency 42.

### 2.3 N7-methylguanosine (m7G) (Fig. 1c)

m7G, the methylation of the seventh nitrogen atom on guanine in RNAs, can be categorized into m7G cap modifications and internal m7G modifications. As early as 1975, Muthukrishnan *et al.* discovered m7G modifications in the 5'-cap structure of mRNAs in viruses and erythroblast cells 48. The methylation of the guanine cap in mammalian mRNAs is catalyzed by RNA guanine-N7 methyltransferase (RNMT) and RNMT-activating miniprotein 49. The 5' cap modified with m7G can protect RNAs from hydrolysis by nucleases and regulate the stability, nuclear export, splicing, and translation of mRNAs by eIF4E and cap-binding complexes 50-53. As the “writer” of internal m7G modifications in mRNAs, the METTL1-WDR4 complex mainly catalyzes the m7G modifications in 5' UTRs, CDS, and 3' UTRs 54. The m7G modifications within mRNA have been shown to promote the translation process 54. Quaking proteins have been identified as readers of mRNA internal m7G modifications. These proteins shuttle m7G-modified transcripts into stress granules to enhance mRNA stability and reduce translational efficiency 55. To date, the erasers responsible for removing m7G modifications have yet to be discovered. Identifying these erasers is essential for elucidating the comprehensive regulatory mechanisms through which m7G modifications influence mRNAs.

### 2.4 N4-acetylcytosine (ac4C) (Fig. 1d)

ac4C is an acetylation modification that occurs on the fourth nitrogen atom of cytosine 56, and its abundance is much lower than other RNA modifications 57. Early research on ac4C primarily focused on tRNAs and rRNAs in bacteria and fungi 58, 59. In 2018, ac4C was first discovered in the mRNAs of HeLa cells 60. The comprehension of ac4C remains elementary, and so far, NAT10 stands as the sole identified writer of ac4C, primarily localized within the nucleus 61, 62. It is noteworthy that ac4C in different regions of mRNAs has diverse effects on translation. ac4C modifications in CDS significantly enhance the stability of mRNAs, leading to improved translation efficiency 60. Furthermore, ac4C modifications at the codon wobble position were found to facilitate the recognition of cognate tRNAs to mRNAs, resulting in the upregulation of translation efficiency 63. Conversely, acetylation in the 5' UTR can suppress translation initiation 64. Due to limited research on ac4C, the identification of ac4C erasers and readers has not yet been reported. Notably, remodelin has been identified as an inhibitor of NAT10 65.

### 2.5 5-methylcytosine (m5C) (Fig. 1e)

The m5C modifications of RNAs involve the methylation of the fifth carbon atom of cytosine 66. m5C is particularly prominent in tRNAs and rRNAs but relatively rare in mammalian mRNAs, accounting for only 0.02%-0.09% of the total cytosine content 67, 68. The majority of m5C modifications in mRNAs are mediated by NSUN2 and NSUN6, while the remaining modifications are attributed to NSUN4 69-71. Unlike other RNA modifications, the erasers of m5C do not directly remove the methyl group but rather oxidize m5C to products such as 5-hydroxymethylcytosine 72. The erasers of m5C include the ten-eleven translocation (TET) family of demethylases (TET1, TET2, and TET3), as well as ALKBH1, which prevents the binding of m5C reader proteins by oxidizing m5C 73, 74.

Currently identified m5C readers include Aly/REF export factor (ALYREF) 69, YBX1 75, as well as YTHDF2 and SRSF2 76, 77. ALYREF regulates the nuclear export of mRNAs 69, while YBX1 modulates mRNA translation and enhances mRNA stability by recruiting poly(A) binding protein 1 and ELAV-like RNA binding protein 1 to mRNAs 72, 78, 79.

## 3. Role of mRNA modifications in BDs (Fig. 2)

BDs, encompassing diverse bone, joint, and soft tissue issues, are a major disability cause globally, with significant economic implications. Many studies have emphasized that chemical modifications within mRNAs affect the physiological and pathological processes of cells involved in BDs, thus playing a crucial role in BDs, as shown in Table 1. This review introduces the important roles of mRNA modifications in the following seven BDs.

### 3.1 Osteoporosis (OP) (Fig. 2a)

OP is a systemic BD resulting from an imbalance in bone cell function characterized by enhanced bone fragility, decreased bone density, and degradation of bone tissue microstructure 1, 80. OP can be divided into two categories: primary and secondary, with the primary type being more common 81. Primary OP comprises mainly senile OP and postmenopausal OP, while secondary OP can be triggered by a variety of factors, including diseases, medications, and nutritional imbalances 82, 83. As a common metabolic BD, OP poses a major challenge to global public health 84.

Downregulation of METTL14 in the bone tissues of OP patients and OVX mice is believed to promote osteoclast differentiation and inhibit osteoblast differentiation. Increased osteoclast differentiation is attributed to decreased *SIRT1* mRNA stability and increased *NFATc1* mRNA stability, both of which result from reduced m6A modification 85, 86. Deng *et al.* demonstrated that overexpression of METTL14 inhibits osteoclast differentiation by enhancing the stability of *GPX4* mRNA, further reinforcing the concept that METTL14 deficiency facilitates osteoclast differentiation 87. METTL14 inhibits osteoclastogenesis and bone resorption via enhancing *GPX4* stability through an m6A-HuR dependent mechanism. Additionally, the inhibition of osteoblast differentiation is linked to the reduced stability of *Beclin-1* and* SMAD1* mRNA by downregulating METTL14 6, 88. Overexpression of METTL14 promotes osteoblast differentiation by enhancing the stability of *TCF1* mRNA, supporting the notion that METTL14 deficiency inhibits osteoblast differentiation 89.

Upregulated METTL3 in the bone tissues of OP patients and OVX mice is deemed to enhance osteoclast differentiation and bone resorption 90. This viewpoint is further supported by the finding that silencing METTL3 inhibits osteoclast differentiation 91. Mechanistically, METTL3 promotes osteoclast differentiation by stabilizing *CHI3L1* mRNA, while its silencing reduces *Atp6v0d2* expression and impairs *Traf6* nuclear export, leading to decreased osteoclast differentiation 90, 91. However, METTL3 also facilitates osteoblast differentiation and mitigates osteoporosis progression by enhancing the translation efficiency of *Pth1r* mRNA 92. The reported data suggest that the upregulation of METTL3 promotes osteoblast and osteoclast differentiation. High levels of METTL3 in bone tissue ultimately result in low bone mass, potentially due to the stronger promotion of osteoclast differentiation.

FTO is upregulated during osteoblast differentiation and is thought to promote this process by reducing the stability of *PPARG* mRNA and increasing the stability of protective protein mRNAs, such as *Hspa1a* 93, 94. Additionally, downregulation of FTO promotes osteoclast differentiation in diabetes-induced OP, further indicating that upregulated FTO plays a crucial role in counteracting OP 95. ALKBH5, another m6A eraser, is also upregulated during osteoblast differentiation 96. Knockdown of ALKBH5 inhibits osteoblast differentiation by reducing the stability of *Runx2* mRNA, further confirming the essential role of ALKBH5 upregulation in osteoblast differentiation 96.

Studies have demonstrated that the m6A readers play crucial roles in OP prevention by promoting osteoblast differentiation and inhibiting osteoclast differentiation, respectively. Specifically, YTHDF1 enhances the translation of *Zfp839*, which promotes osteoblast differentiation through its interaction with *Runx2* 97. Additionally, YTHDC1 inhibits osteoclast differentiation by stabilizing *PTPN6* mRNA in an m6A-HUR-dependent manner 98. Moreover, aberrant expression of METTL3 and METTL14 leads to abnormal m6A modification levels on mRNAs related to osteoclast and osteoblast differentiation. This affects the recognition of these m6A-modified mRNA by m6A readers, resulting in abnormal biological effects by readers. Specifically, in OP, the downregulation of METTL14 reduces m6A modifications on *Beclin-1* and *SMAD1* mRNA, weakening the recognition of these mRNAs by IGF2BPs and the stabilizing effects on mRNAs. This leads to decreased stability of *Beclin-1* and *SMAD1* mRNAs, thereby inhibiting osteoblast differentiation 6, 88. Additionally, supplementation of METTL14 increases m6A modifications on *NFATc1*, enhancing its recognition by YTHDF2 and diminishing its stabilization, ultimately suppressing osteoclast differentiation 85. The upregulation of METTL3 in osteoporosis increases m6A modifications on *Atp6v0d2*, facilitating its recognition by YTHDF2, which reduces its stability. This process results in the differentiation of smaller osteoclasts with enhanced bone resorption activity 91.

The downregulation of the ac4C writer NAT10 and the m5C reader YBX1 contributes to the pathogenesis of OP by inhibiting osteoblast differentiation. Specifically, downregulated NAT10 reduces the stability of *RUNX2* mRNA, leading to suppressed osteoblast differentiation 7. Ybx1 downregulation diminishes the stability of *BMP4* mRNA and blocks its release from the endothelium, further inhibiting osteoblast differentiation 8. Additionally, research on m1A modifications in OP remains limited, with preliminary findings identifying 28 m1A-SNPs associated with bone mineral density 99.

### 3.2 Osteoarthritis (OA) (Fig. 2b)

OA is the most common joint disease that involves damage to the articular cartilage, alteration of the subchondral bone, and affecting the ligaments and muscles surrounding the joint 100. Age serves as the primary risk factor for OA, with gender, genetics, and obesity contributing as well 101. OA has been shown to increase the risk of diabetes in older adults, hinder the prevention of serious complications in diabetic patients, and negatively impact both cardiovascular and mental health in the elderly 102-105. Notably, chondrocytes and FLSs play pivotal roles in OA, and the chemical modifications of mRNAs can affect their physiological and pathological processes.

Upregulation of METTL3 is considered to be involved in the progression of OA. Elevated METTL3 levels not only promote chondrocyte pyroptosis by increasing *NEK* mRNA expression but also impair autophagy in OA-FLSs by reducing the stability of *ATG7* mRNA 106, 107. Additionally, METTL3 downregulation has been shown to inhibit chondrocyte ferroptosis and alleviate OA progression, further underscoring the critical role of upregulated METTL3 in OA pathogenesis 108. WTAP, another m6A writer, is upregulated in clinical osteoarthritic cartilage and TNF-α-induced chondrocytes. Elevated WTAP expression causes extracellular matrix degradation, inflammation, and oxidative stress in osteoarthritic chondrocytes by reducing *FRZB* levels and activating the Wnt/β-catenin pathway 109.

Aberrant expression of m6A erasers and readers significantly influences the progression of OA by modulating complex biological processes, such as senescence and inflammatory responses. Specifically, downregulation of ALKBH5 in OA enhances the stability of *CYP1B1* mRNA, which leads to mitochondrial dysfunction and accelerates the aging of mesenchymal stem cells (MSCs) 110. Moreover, upregulation of IGF2BP3 in OA promotes macrophage polarization and the expression of inflammatory cytokines 111. In patients with OA, the expression levels of FTO and IGFBP2 are also elevated, while YTHDF2 expression is decreased 112, 113. However, the precise mechanisms by which these molecules contribute to OA pathogenesis remain to be further elucidated.

In recent years, the role of m7G has attracted attention in OA research. Studies have shown that the m7G reader EIF4E2 is significantly upregulated in OA synovial tissues, while the m7G writer METTL1 is notably downregulated 114, 115. These findings suggest a potential involvement of m7G in OA pathogenesis, although the specific mechanisms remain largely unexplored and require further investigation.

### 3.3 Rheumatoid arthritis (RA) (Fig. 2c)

RA is a chronic inflammatory autoimmune disease characterized by immune cell infiltration, synovial hyperplasia, and destruction of the articular cartilage and bone 116, 117. Patients with RA can suffer from progressive joint ankylosis, destruction, deformity, and disability if left untreated 117. Approximately 1% to 2% of the world's population suffers from RA, with women at a higher risk of developing the disease 118.

The m6A writers play critical roles in the pathogenesis of RA by regulating inflammation and FLS activation. Studies have demonstrated that both METTL14 expression and m6A modifications are significantly downregulated in the peripheral blood mononuclear cells of RA patients 9. This reduction enhances the stability of *TNFAIP3* mRNA, leading to the increased expression of inflammatory cytokines, such as IL-6 and IL-17 9. However, another study indicated that METTL14 expression was upregulated in synovial tissues, which stabilized *LASP1* mRNA and exacerbated FLS activation and inflammation via the LASP1/SRC/AKT signaling pathway 119. These findings suggest that the expression of modified regulators may vary across different organ tissues in the same disease, and that the specific mechanisms need further investigation. As another m6A writer, METTL3 has been implicated in promoting oxidative stress and inflammation in arthritic tissues by destabilizing *TTC4* mRNA 120. The downregulation of METTL3 inhibits the ICAM2/PI3K/AKT/p300 signaling pathway, effectively slowing RA progression and further supporting the critical role of METTL3 in RA pathogenesis 121.

Aberrant m6A and m5C modifications are pivotal in the pathogenesis of RA. Specifically, upregulation of the m6A eraser ALKBH5 significantly promotes the proliferation, migration, and invasion of RA FLSs by increasing the stability of *JARID2* mRNA 122. On the other hand, elevated expression of the m5C writer NSUN2 in RA decreases the stability of* SFRP1* mRNA, leading to activation of the Wnt/β-catenin signaling pathway and thereby accelerating RA progression 10.

### 3.4 Osteosarcoma (OS) (Fig. 2d)

OS, the primary bone tumor mainly affecting children and adolescents, is best treated with a combination of surgery and chemotherapy 123-125. Although chemotherapy has raised the five-year survival rate to nearly 80%, the overall survival rate remains in the 60%-70% range due to challenges such as metastasis, drug resistance, and high recurrence rates 126-128.

The abnormal elevation of m6A writers has been reported to promote the proliferation of OS cells and the formation of drug resistance. Specifically, upregulation of WTAP reduces the stability of *HMBOX1* mRNA and activates the PI3K/AKT signaling pathway, thereby promoting tumor cell proliferation 129. Additionally, overexpression of METTL14 has been shown to increase the translation efficiency of *MN1* mRNA, leading to all-trans retinoic acid (ATRA) chemotherapy resistance and accelerating tumor progression 11. Furthermore, the upregulation of METTL3 stabilizes *ATG5* mRNA, enhancing autophagy, which is another key mechanism driving OS progression 130. Additionally, clinical data have revealed that the downregulation of the m6A eraser ALKBH5 contributes to OS development 131. This finding is further supported by subsequent experiments, where overexpression of ALKBH5 effectively inhibits OS progression by stabilizing *SOCS3* mRNA and activating the STAT3 signaling pathway 131.

Upregulation of the m5C writer NSUN2 and the ac4C writer NAT10 is implicated in tumor progression through the regulation of cellular metabolic mechanisms. Specifically, elevated NSUN2 expression significantly enhances fatty acid metabolism in tumor cells by stabilizing *FABP5* mRNA 12. Notably, chemotherapy-induced downregulation of NSUN2 expression can effectively trigger apoptosis in tumor cells, further reinforcing the link between NSUN2 upregulation and poor prognosis in OS 132. Additionally, increased NAT10 expression promotes glycolysis in tumor cells by stabilizing *YTHDC1*, *PFKM*, and *LDHA* mRNA, thus providing essential energy support for tumor growth 13.

Current research on m7G and m1A modifications in OS remains in the early exploratory stages. Upregulation of the m7G writer complex METTL1/WDR4 in OS has been shown to enhance the m7G methylation of tRNA, thereby promoting OS progression by increasing the translation efficiency of oncogenic mRNAs 133. However, it has not yet been demonstrated that METTL1/WDR4 directly modifies mRNA to contribute to OS development. Additionally, the m1A writer TRMT10C has been observed to potentially promote epithelial mesenchymal transition and increase sensitivity to chemotherapeutic drugs in tumor cells by regulating the expression of *ACAT1* 134.

### 3.5 Others (Fig. 2e-f)

Fractures represent disruptions in the structural integrity and continuity of bone, often resulting from trauma or pathological conditions 135. Since about 10% of fractures failed to heal properly under traditional therapy (surgery and conservative treatment), it is important to explore the roles of mRNA modifications in fracture healing to find new therapeutic strategies 136.

METTL3 expression is markedly downregulated during the early phases of fracture healing and is thought to facilitate this reparative process 137. *In vivo* studies demonstrate that local administration of the METTL3 plasmid at the fracture site significantly delays healing, reinforcing the notion that METTL3 downregulation is conducive to fracture repair 137. Mechanistic investigations have revealed that METTL3 impairs osteoblast differentiation by inhibiting the maturation of *miR-7212-5p* 137. However, whether METTL3 exerts its inhibitory effects on fracture healing directly through m6A-mediated mRNA regulation remains to be elucidated. Concurrently, the upregulation of YTHDF1 during osteoblast differentiation appears to enhance fracture healing, potentially through the activation of autophagy signaling pathways 14.

AS is a chronic rheumatic disease characterized by persistent inflammation and heterotopic bone formation 138. Clinically, it presents as inflammation at the points of attachment, primarily affecting the spine and sacroiliac joints 139. The disease is more prevalent among young and middle-aged males, increasing the risk of spinal skeletal dysfunction and disability 140, 141. The etiology of AS is complex, involving environmental triggers, infections, genetic susceptibility, and immune dysregulation 142, 143. Recent studies have identified the roles of m6A and ac4C modifications in AS pathogenesis. Specifically, the downregulation of METTL14 has been shown to destabilize *FOXO3a* mRNA, leading to impaired autophagy and exacerbated inflammation 144. Concurrently, decreased METTL14 levels enhance the migratory capacity of MSCs by increasing the stability of *ELMO1* mRNA 145. The restoration or overexpression of METTL14 following therapeutic intervention further underscores the critical role of METTL14 downregulation in AS development 144. Additionally, a significant reduction in FTO expression has been observed in interspinous ligament samples from patients with AS, although the precise mechanism by which FTO contributes to AS pathology remains to be elucidated 146. Moreover, existing research has established a close association between reduced NAT10 expression and AS disease activity, and NAT10 levels can be used to improve the sensitivity and specificity of early AS diagnosis 147.

OM, an inflammatory BD primarily caused by bacterial, fungal, and mycobacterial infections, typically emerges following bone injury or vascular insufficiency 148-150. In bone tissue, limited blood flow and the presence of bacterial biofilms impede antibiotic delivery, making treatment challenging 151, 152. METTL3 is significantly upregulated in both OM animal models and in bone marrow from OM patients, and is believed to play a role in the inflammatory response 153, 154. This viewpoint is further supported by findings that inhibition of METTL3 with STM2457 in OM cell models leads to a marked reduction in the release of IL-6 and TNF-α 153. Additionally, the deletion of the m6A eraser FTO in Staphylococcus aureus-exposed BMSCs has been shown to induce ferroptosis by stabilizing *MDM2* mRNA 155.

## 4. RNA modification-based therapeutics for BDs (Fig. 3)

Therapeutic strategies based on mRNA modifications refer to the modulation of the chemical modifications of mRNAs to effectively inhibit the occurrence and progression of diseases. As shown in Table 2. These strategies can regulate cell differentiation, senescence, and death while suppressing inflammatory responses, ultimately leading to significant improvements in the occurrence and progression of BDs.

### 4.1 Regulating bone homeostasis in OP and fractures (Fig. 3a-b)

Osteoblasts are derived from MSCs in bone marrow and blood 156. Stimulating MSCs to migrate and differentiate into osteoblasts, as well as enhancing their ability to synthesize the bone matrix, is key to treating OP and fractures 157.

Numerous studies have demonstrated that the regulation of m6A deposition in mRNAs plays a critical role in enhancing osteoblast differentiation. Injecting LV-METTL14 can enhance the m6A level and stability of *SIRT1*, *SMAD1*, and *Beclin-1* mRNA, in turn promoting the osteogenic differentiation of BMSCs and alleviating bone loss in OVX mice 6, 86, 88. Transplantation of BMSC sheets overexpressing the m6A reader YTHDF1 around the fracture site has been shown to enhance autophagy, thus promoting osteoblast differentiation and ultimately facilitating fracture healing in rats 14.

Emerging evidence suggests that ac4C and m5C are also important targets in treatment strategies aimed at promoting osteoblast differentiation. It has been reported that intramuscular injection of adenovirus overexpressing NAT10 can upregulate the acetylation and stability of *RUNX2* mRNA, which enhances osteoblast differentiation and increases the trabecular volume fraction and trabecular number in OVX mice 7. Traditional Chinese medicine, Mijiao, has been shown to increase the stability and expression of *RUNX2* by upregulating NAT10, which further improves osteoblast differentiation and mitigates bone loss in OVX mice 158. In addition, the m5C reader YBX1 can be upregulated by injection of sciadopitysin, resulting in increased vascular-dependent osteogenic differentiation via enhancing the stability of *BMP4* mRNA 8.

Osteoclasts, originating from mononuclear/macrophage lineage progenitor cells, play a crucial role in maintaining bone homeostasis by mediating bone resorption 159, yet excessive activation of osteoclasts can lead to pathological bone loss 160. In such cases, inhibiting osteoclast differentiation through the regulation of m6A serves as a strategy for treating OP. For example, injection of METTL14 exosomes into the bone marrow cavity or intravenous administration of LV-METTL14 has been shown to reduce the stability of *NFATc1* mRNA and increase the stability of *SIRT1* mRNA, thus effectively inhibiting osteoclast differentiation and alleviating bone loss in OVX mice 85, 86.

### 4.2 Inhibiting inflammatory response, cell loss, and cellular senescence in OA and OM (Fig. 3c-d)

Inflammation, cellular senescence, and cellular damage are common causes of the development of BDs, such as OA and OM 161, 162. Regulating these pathological processes through the modulation of m6A serves as a therapeutic strategy for BDs. For example, intra-articular injection of AAV-siWTAP can improve the OARSI score in a mouse model of OA 109. According to X *et al.*, the downregulation of WTAP reduced the m6A modifications of *FRZB* mRNA, leading to increased *FRZB* expression, inhibiting the Wnt/β-catenin pathway and ultimately suppressing the inflammatory cascade in OA 109. In addition, an intraperitoneal injection of STM2457 (a METTL3 inhibitor) in an OM mouse model effectively suppresses inflammation by downregulating *MyD88* and NF-κB-related inflammatory molecules in macrophages 153.

Regarding the suppression of cellular senescence, intra-articular injection of MSCs overexpressing ALKBH5 attenuates many types of cellular senescence and reduces inflammation induced by senescent cells, which in turn alleviates cartilage defects and OARSI scores in OA mice 110. The increased expression of ALKBH5 facilitates *CYP1B1* mRNA degradation via m6A demethylation, thus attenuating senescence in MSCs 110. Additionally, intra-articular injection of METTL3 siRNA can increase the stability of *ATG7* mRNA, leading to upregulation of autophagy in OA-FLSs, suppression of MSC senescence, and improvement of cartilage degradation 107. The pyroptosis of chondrocytes and the expression of inflammatory factors in OA mice can be inhibited by the injection of sh-METTL3 106. Xiong *et al.* demonstrated that the m6A modifications of *NEK7* mRNA were reduced by the downregulation of METTL3, leading to decreased *NEK7* expression and reduced chondrocyte pyroptosis 106. Furthermore, the intravenous injection of lentiviruses carrying pcDNA-FTO vectors reduces the methylation and stability of *MDM2* mRNA, further downregulating the MDM2/TLR4/SLC7A11 signaling pathway, which subsequently inhibits ferroptosis in BMSCs and reduces histopathological damage in bone 155.

### 4.3 Others (Fig. 3e-f)

It has been reported that inhibiting the proliferation and migration of FLSs while promoting their apoptosis can reduce synovial hyperplasia and cartilage degeneration in RA 163, 164. Intraperitoneal injection of artemisitene in an RA mouse model inhibits METTL3, which in turn reduces the expression of *ICAM2* mRNA, ultimately preventing the migration and invasion of RA-FLSs and inducing apoptosis 121. Intra-articular injection of sh-METTL14 in rat models of RA has been found to prevent the activation, proliferation, and invasion of FLSs by reducing the methylation and stability of *LASP1* mRNA, thus inhibiting the LASP1/SRC/AKT signaling axis 119. In addition, the proliferation, migration, and invasion of FLSs are inhibited by intra-articular injection of sh-ALKBH5, which reduces m6A modification levels and the stability of *JARID2* mRNA 122.

Suppressing the proliferation, metastasis, and drug resistance of OS represents a central challenge in the treatment of this disease. Wang *et al.* reveal that injecting Spautin-1 into mice bearing xenografts effectively inhibits the USP13-enhanced stabilization of METTL3, subsequently reducing the stability of *ATG5* mRNA and thereby curbing tumor proliferation and metastasis 130. Additional researches indicate that knockdown of METTL14, NAT10, and NSUN2 in OS cell lines significantly inhibits tumor growth in nude mice 11-13. Overexpression of ALKBH5 has also been found to suppress the proliferation of U2OS cells in nude mice 131. Moreover, Li *et al.* further elucidate that knocking down METTL14 and NSUN2 in OS cells can respectively potentiate the inhibitory effects of ATRA and doxorubicin on tumors, thereby suppressing tumor drug resistance 11, 132.

Abnormally increased osteogenic differentiation of MSCs has been shown to play a significant role in pathological bone formation in AS 165, and inhibiting this differentiation has been identified as a strategy to alleviate spinal joint stiffness. Xie *et al.* reported that the injection of AV-*ELMO1* into the tail vein counteracted METTL14-induced upregulation of *ELMO1* expression, thus ameliorating heterotopic ossification 145.

## 5. Conclusions and future perspectives

Scientists have made significant progress in revealing the epigenetic mechanisms underlying the occurrence and progression of BDs. As an important component of epigenetics, mRNA modifications have emerged as new focuses and novel therapeutic targets against BDs. In this review, we have summarized the regulatory mechanisms of five mRNA modifications and explored their involvement in the occurrence and development of BDs. Current research has highlighted that the cooperation between writers and erasers achieves dynamic reversibility of modifications, and that different modification types, modification sites, and readers will have different effects on the processes of mRNA export, translation, and degradation. We found that abnormal mRNA modification levels were involved in the development of BDs in multiple aspects. Abnormal modifications can affect mRNA stability and translation efficiency, disrupt the balance between osteogenic and osteoclastic differentiation, promote inflammation and cytopathia, and induce metabolic reprogramming and drug resistance in tumors. In addition, targeting mRNA modification regulators shows promise in diagnosing and treating BDs. Taking m6A modification as an example, Deng *et al.* demonstrated that METTL14 is downregulated in serum samples from postmenopausal women with OP and positively correlates with bone mineral density 87. This finding suggests that METTL14 has potential as a diagnostic biomarker for OP. Furthermore, animal studies have confirmed that modulating abnormal levels of m6A regulators, such as through viral injection methods, can effectively treat OP. Therefore, developing safe and clinically validated drugs targeting m6A modification regulators holds significant clinical value for the treatment of OP.

The targetability and safety of mRNA-modified therapeutic strategies need to be improved. Viral vectors, agonists, and inhibitors have achieved good therapeutic effects in animal models by regulating mRNA modifications. However, mRNA modification regulators may regulate multiple mRNAs simultaneously, which can lead to the occurrence of other diseases while treating BDs. Moreover, mRNA modification regulators can exert opposing effects when acting on different cell types. Therefore, accurately targeting diseased cells and specific mRNAs in BDs is essential for improving the therapeutic efficacy of BDs and avoiding the occurrence of side effects. Nanomaterials, particularly bone-targeted nanotechnology, may be ideal candidates for achieving this goal 166. Furthermore, given the critical role of mRNA modifications in biological processes, further exploration of unknown mRNA modification regulators—such as specific readers of m1A—and their molecular mechanisms underlying biological effects is essential for advancing our understanding of this field. Building on this foundation, in-depth investigations into mRNA modifications associated with BDs, as well as the mechanisms by which pathogenic factors lead to dysregulation of these modification regulators, hold significant scientific value and clinical relevance. Although this review discusses mRNA modifications in some BDs, whether mRNA modifications are involved in the development of certain metabolic BDs, such as rickets, hyperthyroidism, and hypothyroidism, and their underlying mechanisms, remains to be further investigated.

Future studies will aim to elucidate the complex networks and mechanisms of action between specific lncRNAs and DNA/RNA modifying enzymes in BDs. In these future studies, two models of modification synergistic effects are particularly worthy of further elucidation. The first model involves one type of mRNA modification regulating the expression of the regulatory factors of another mRNA modification, thereby influencing the occurrence and progression of BDs through the latter modification. The other modification interaction is reflected in different mRNA modifications jointly regulating the same mRNA, collectively determining the progression of the disease. These complex networks and mechanisms will go beyond mere descriptions of associations to portray deep molecular mechanism atlases. The discovery of these epigenetic regulatory mechanisms may lead to potential therapeutic approaches. As our understanding of interactions deepens, epigenetic biomarkers may be utilized for the early diagnosis, prognostic assessment, and monitoring of the therapeutic response in BDs. Future treatments for BDs are likely to evolve in a more personalized direction. By targeting specific aberrant mRNA modifications in individual patients, interventions are expected to improve the efficacy and precision of treatment.

## Figures and Tables

**Figure 1:**
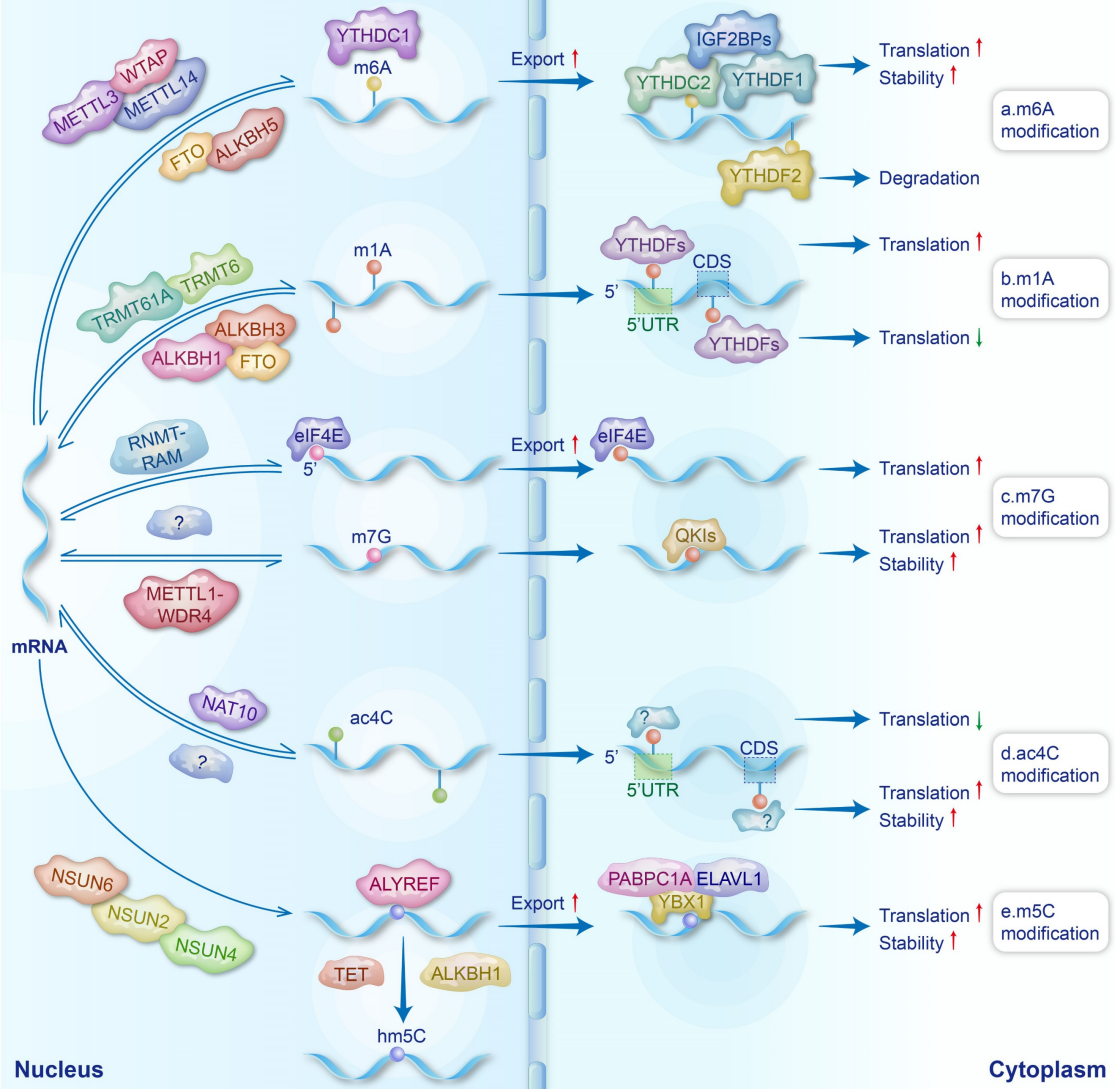
** The regulatory processes of mRNA modifications.** Formation and erasure mechanisms of mRNA modifications, along with their impacts on mRNA.

**Figure 2:**
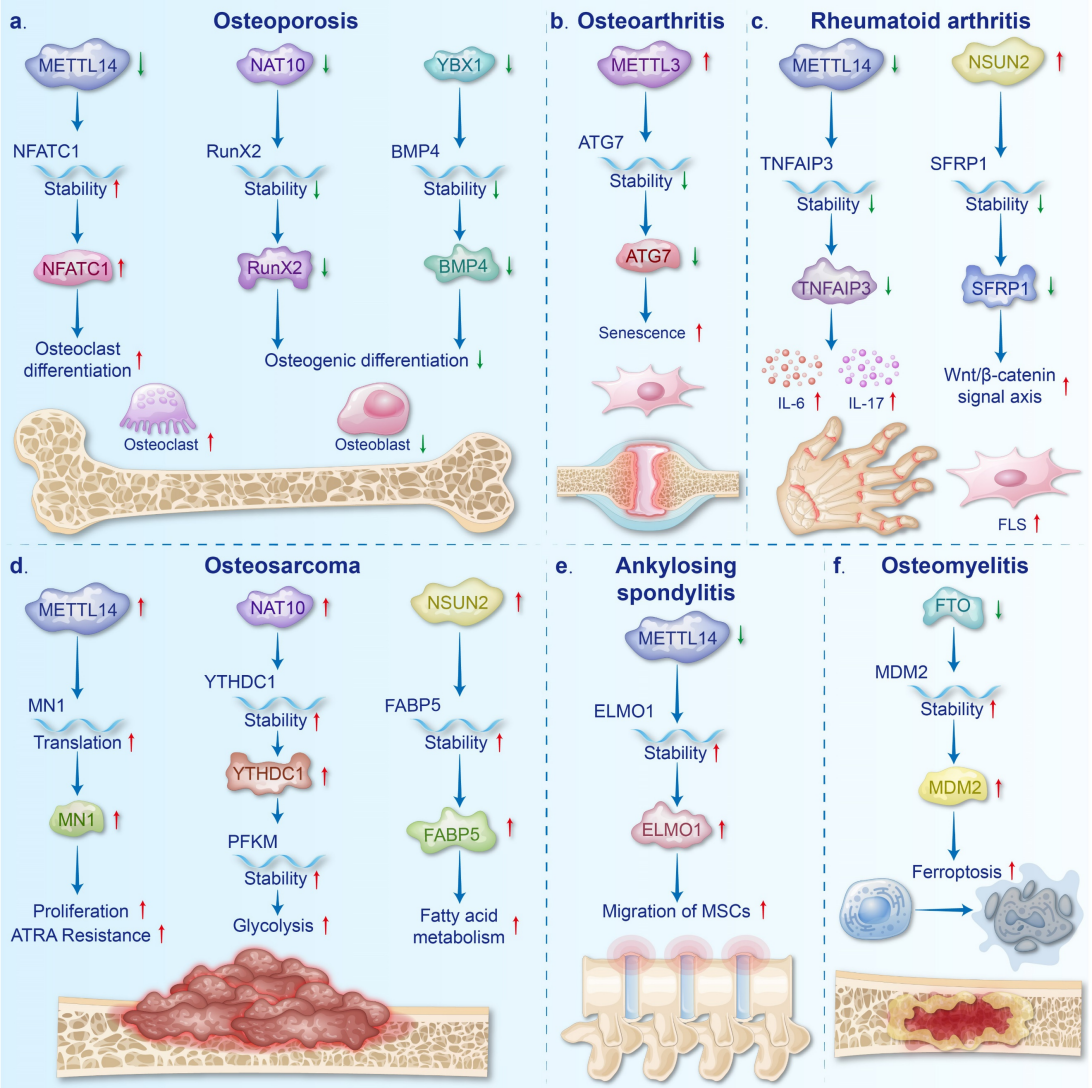
** Mechanisms of mRNA modifications in the BDs.** a. Abnormalities of METTL14 (m6A), NAT10 (ac4C) and YBX1 (m5C) affected the mRNA stability of NFATC1, RUNX2 and BMP4, respectively. These processes contributed to disorders of osteoclast differentiation and osteoblast differentiation leading to OP. b. The mRNA stability of ATG7 is reduced by the upregulation of METTL3 (m6A), which contributes to the senescence of FLSs in OA. c. Dysregulation of METTL14 (m6A) and NSUN2 (m5C) reduced mRNA stability of TNFAIP3 and SFRP1, respectively, and promoted inflammation and proliferation of FLS in RA. d. The mRNA translation of MN1 and the mRNA stability of YTHDC1 and FABP5 were regulated by METTL14 (m6A), NAT10 (ac4C), and NSUN2 (m5C), respectively, which resulted in the progression of OS. e. The downregulation of METTL14 (m6A) enhanced the mRNA stability of ELMO1, and promoted the migration and osteogenesis differentiation of MSCs in AS. f. mRNA stability of MDM2 was increased by FTO (m6A) downregulation, which promoted BMSCs ferroptosis in OM.

**Figure 3:**
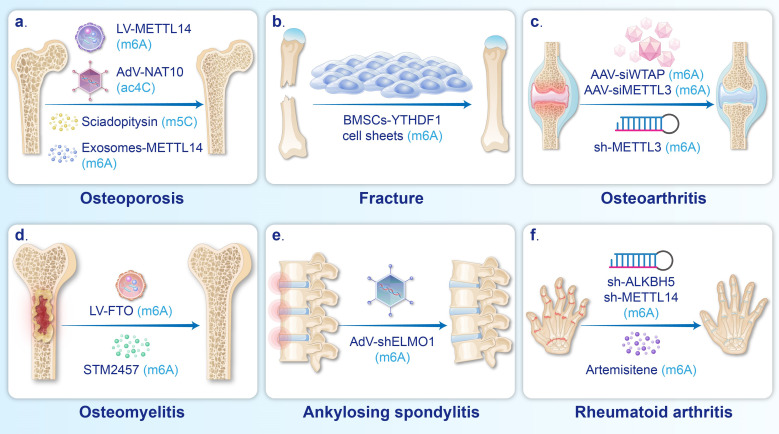
** Therapeutic strategies for BDs based on mRNA modifications.** a. AdV-NAT10, LV-METTL14, Sciadopitysin, and METTL14 exosomes were used to alleviate OP. b. Cell sheets of BMSCs overexpressing YTHDF1 promoted fracture healing. c. Intra-articular injection of AAV-siWTAP, AAV-siMETTL3, and sh-METTL3 could mitigate the progression of OA. d. OM can be relieved by injection of LV-FTO and STM2457. e. Injection of AdV-shELMO1 could alleviated ectopic ossification in AS. f. The progression of Rheumatoid arthritis could be suppressed by the treatment of Artemisitene, sh-METTL14, and sh-ALKBH5.

**Table 1:** mRNA modifications in bone diseases.

Modification	Regulator	Disease	Expression (sample)	Mechanism/function	Refs.
m6A	METTL14	osteoporosis	Downregulated (human and mouse)	METTL14-m6A-*NFATc1*, *SIRT1*, *Beclin-1*, *SMAD1*, *GPX4* and *TCF1/RUNX2* mRNA modulating osteoblast and osteoclast differentiation	6,85-89
		ankylosing spondylitis	Downregulated (human)	METTL14-m6A-*FOXO3a* mRNA modulating autophagy activity and inflammation	144
				METTL14-m6A-*ELMO1* mRNA modulating the directed migration of MSCs	145
		rheumatoid arthritis	Downregulated (human)	METTL14-m6A-*TNFAIP3* mRNA modulating the progression of inflammation	9
			Upregulated (rat)	METTL14-m6A-*LASP1* mRNA modulating the activation of FLSs and related inflammatory reactions	119
		osteosarcoma	Upregulated (human)	METTL14-m6A-*MN1* mRNA modulating tumor progression and ATRA chemotherapy resistance	11
	METTL3	osteoporosis	Upregulated (mouse)	METTL3-m6A-*CHI3L1*,*Atp6v0d2*,*Traf6* and* Pth1r* modulating osteoclast differentiation, bone resorption and the osteogenic differentiation of BMSCs	90-92
		rheumatoid arthritis	ATT inhibits METTL3 (mouse)	METTL3-m6A-*ICAM2* mRNA modulating apoptosis of RA-FLS cells, inhibiting proliferation, migration, and invasion	121
			—— (mouse)	METTL3-m6A-*TTC4* mRNA modulating inflammation and oxidative stress	120
		osteoarthritis	Upregulated (human and rat)	METTL3-m6A-*ACSL4*,*NEK7* mRNA regulating chondrocyte ferroptosis and pyroptosis	106, 108
				METTL3-m6A-*ATG7* mRNA regulating autophagy and senescence in FLSs	107
		osteomyelitis	Upregulated (human and rat)	——	153, 154
		osteosarcoma	Upregulated (human)	METTL3-*ATG5* mRNA modulating oncogenic autophagy	130
	WTAP	osteoarthritis	Upregulated (human)	WTAP-m6A-*FRZB* mRNA regulating extracellular matrix degradation, inflammatory response, and oxidative stress in osteoarthritis chondrocytes	109
		osteosarcoma	Upregulated (human)	WTAP-m6A-*HMBOX1* mRNA regulating osteosarcoma growth and metastasis via PI3K/AKT pathway	129
	FTO	osteoporosis	Downregulated (human)	FTO-m6A-*PPARG* and protective protein mRNAs modulating osteoblast differentiation and bone formation; safeguarding osteoblasts from genotoxic	93,94
		osteomyelitis	Downregulated (rat)	FTO-m6A-*MDM2* mRNA modulating SA-triggered ferroptosis in BMSCs	155
		osteoarthritis	Upregulated (human)	——	112
	ALKBH5	osteoporosis	Upregulated in osteogenic process (rat)	ALKBH5-m6A-*Runx2* mRNA modulating osteoblast differentiation	96
		osteoarthritis	Downregulated (mouse)	ALKBH5-m6A-*CYP1B1* mRNA, leading to mitochondrial dysfuncton and promoting MSC senescence	110
		rheumatoid arthritis	Upregulated (human)	ALKBH5-m6A-*JARID2* mRNA modulating the proliferation, migration, and invasion of RA FLSs	122
		osteosarcoma	Downregulated (human)	ALKBH5-m6A-*SOCS3* mRNA modulating cell proliferation and apoptosis	131
	YTHDF1	osteoporosis	Downregulated (human)	YTHDF1-m6A-*Zfp839* mRNA modulating BMSC osteogenesis	97
		osteoarthritis	Downregulated (human)	——	113
	YTHDC1	osteoporosis	Downregulated (human and mouse)	YTHDC1-m6A-HUR-*PTPN6* mRNA modulating osteoclast differentiation	98
	IGFBP2	osteoarthritis	Upregulated (human)	——	113
ac4C	NAT10	osteoporosis	Downregulated (human and mouse)	NAT10-ac4C-*RUNX2* mRNA modulating osteogenic differentiation of bone BMSCs	7
		osteosarcoma	Upregulated (human)	NAT10-ac4C-*YTHDC1*mRNA-m6A*-LDHA/PFKM* mRNA modulating the glycolysis of cancer cells	13
m5C	NSUN2	osteosarcoma	Upregulated (human)	NSUN2-m5C-*FABP5* mRNA and anti-apoptotic mRNA modulating fatty acid metabolism and apoptosis in osteosarcoma cells	12,132
		rheumatoid arthritis	Upregulated (human and rat)	NSUN2-m5C-*SFRP1* mRNA modulating Wnt/β-catenin signaling pathway and proliferation of RA FLS	10
	YBX1	osteoporosis	Downregulated (mouse)	YBX1-m5C-*BMP4* mRNA modulating osteogenic differentiation of BMSCs	8
m7G	METTL1	osteosarcoma	Upregulated (human)	——	133
		osteoarthritis	Downregulated (human)	——	115
	eIF4E2	osteoarthritis	Upregulated (human)	——	114
m1A	TRMT10C	osteosarcoma	——	*ACAT1* was identified as an m1A methylation-related metabolic gene	134

**Table 2:** Therapeutic strategies based on mRNA modifications.

Modification	Disease	Intervention	Target mRNA/axis	Functions/mechanisms	Refs.
m6A	osteoporosis	LV-METTL14, AV-METTL14, METTL14 exosomes injection	*SIRT1*, *SMAD1*, *NFATC1*, *Beclin-1*	Promoting osteogenic differentiation of BMSCs and inhibiting osteoclast differentiation	6,85-87
	fracture	Cell sheets of BMSCs overexpressing YTHDF1	Autophagy signaling pathway	Promoting osteogenic differentiation of BMSCs	14
	osteoarthritis	AAV-siWTAP injection	*FRZB*	Inhibiting inflammation and extracellular matrix degradation, suppressing oxidative stress	109
		sh-METTL3 injection	*NEK7*	Inhibiting chondrocyte pyroptosis and suppressing inflammation	106
		MSCs overexpressing ALKBH5	*CYP1B1*	Mitigating cellular senescence and reducing inflammation	110
		AAV9.HAP-1-si-METTL3 injection	*ATG7*	Suppressing cellular senescence and ameliorated cartilage destruction	107
	osteomyelitis	LV-pcDNA-FTO vector injection	*MDM2/TLR4/SLC7A11* axis	Suppressing iron death	155
		STM2457 injection	METTL3-*MyD88*	Inhibiting METTL3 to alleviate inflammation	153
	rheumatoid arthritis	Artemisitene injection	METTL3-*ICAM2*	Inhibiting METTL3 to impede the migration and invasion of RA-FLSs	121
		sh-ALKBH5 injection	*JARID2*	Inhibiting proliferation, migration, and invasion of RA FLSs	122
		sh-METTL14 injection	*LASP1/SRC/AKT* axis	Inhibiting the activation, proliferation, and invasion of FLSs	119
	ankylosing spondylitis	AdV-shELMO1 injection	*ELMO1*	Counteracting the upregulation of ELMO1 expression caused by METTL14, thereby ameliorating ectopic ossification	145
	osteosarcoma	Spautin-1	*USP13/METTL3/ATG5*	Inhibiting proliferation and metastasis of OS	130
m5C	osteoporosis	sciadopitysin	*YBX1*	Improving osteogenic differentiation of BMSCs	8
ac4C	osteoporosis	AdV-NAT10 injection	*RUNX2*	Promoting osteogenic differentiation of BMSCs	7
		Mijiao	*NAT10*	Promoting osteogenic differentiation of BMSCs by boosting NAT10 expression	158

## Abbreviations

BDs: bone diseases
OP: osteoporosis
RA: rheumatoid arthritis
OS: osteosarcoma
OA: osteoarthritis
OM: osteomyelitis
AS: ankylosing spondylitis
FLSs: fibroblast-like synoviocytes
BMSCs: bone marrow mesenchymal stem cells
MSCs: mesenchymal stem cells
OVX: ovariectomized
m6A: N6-methyladenosine
m1A: N1-methyladenosine
m7G: N7-methylguanosine
ac4C: N4-acetylcytosine
m5C: 5-methylcytosine
3' UTR: 3'untranslated region
5' UTR: 5'untranslated region
CDS: coding sequence
MTC: methyltransferase complex
FTO: fat mass and obesity-associated protein
IGF2BPs: insulin-like growth factor 2 mRNA-binding proteins
hnRNPs: heterogeneous nuclear ribonucleoproteins
eIF: eukaryotic translation initiation factor
TRMT61A: tRNA methyltransferase 61A
TRMT6: tRNA methyltransferase 6
RNMT: RNA guanine-N7 methyltransferase
TET: ten-eleven translocation
ALYREF: Aly/REF export factor
YBX1: Y-Box binding protein 1
BMP4: bone morphogenetic protein 4
ATRA: all-trans retinoic acid
